# Supplementary material for: Epstein-Barr Virus BARF1 Is Expressed in Lung Cancer and Is Associated with Cancer Progression
Source: Cells. 2024 Sep 19;13(18):1578. doi: 10.3390/cells13181578 (PMC11430695; doi:10.3390/cells13181578)
Supplement: Supplementary file 1 [file cells-13-01578-s001.zip › cells-3146469-supplementary.pdf]

SUPPLEMENTARY INFORMATION.

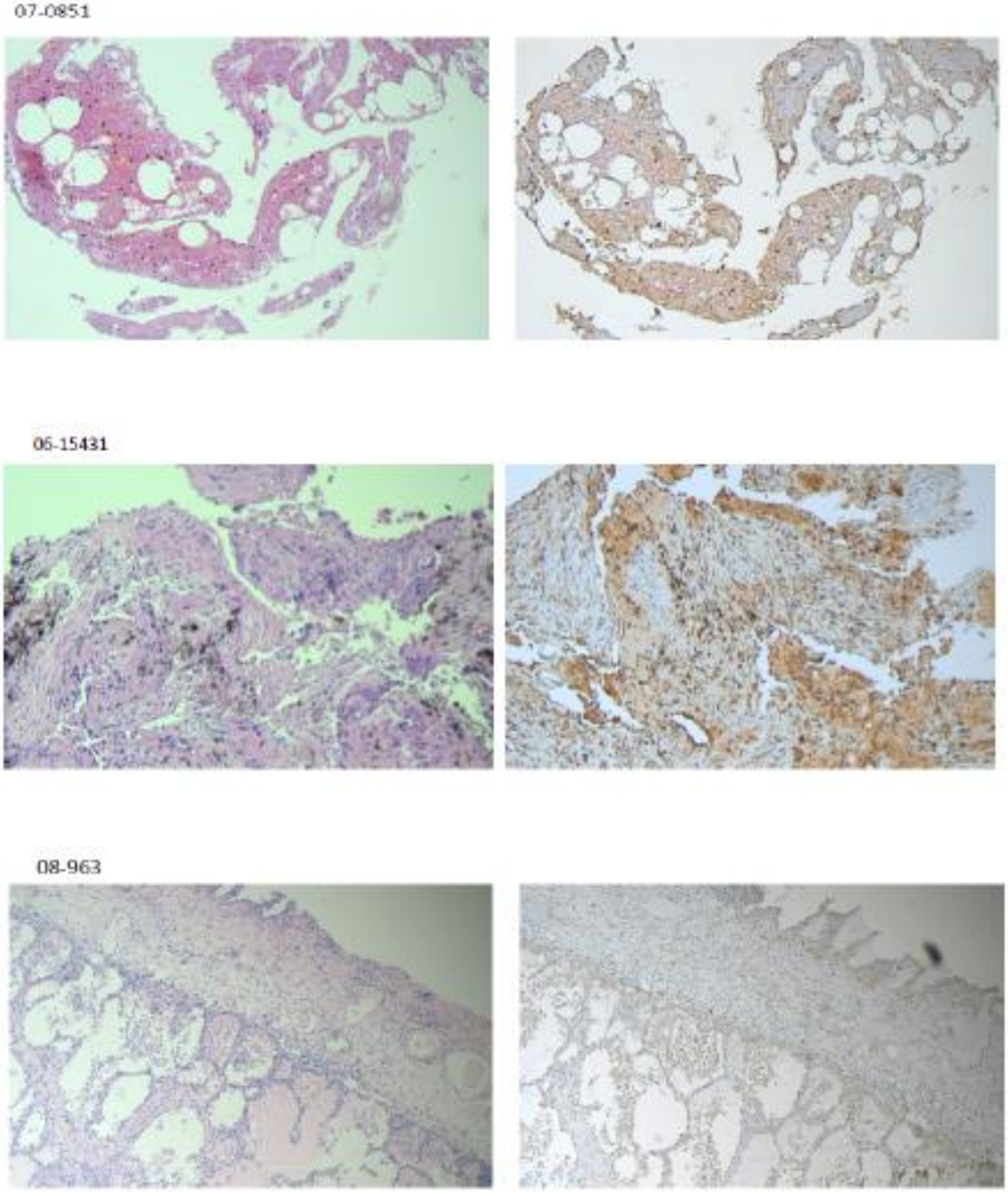

Supplementary figure 1. Immunohistochemistry for EBV-positive lung carcinomas. Three EBV-positive lung cancer specimens are shown. Left: Hematoxylin/Eosin staining (HE); Right: EBNA1 immunostaining.

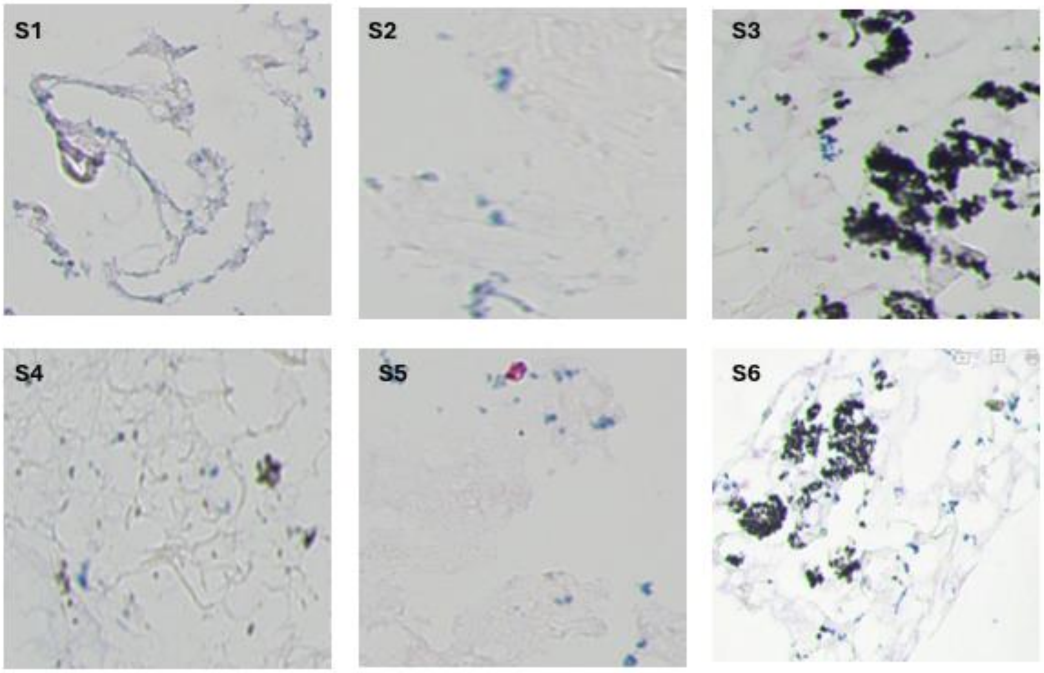

Supplementary figure 2. EBER CIHS for EBV-positive lung carcinomas. S3 and S6 sample present anthracosis and EBER positive.

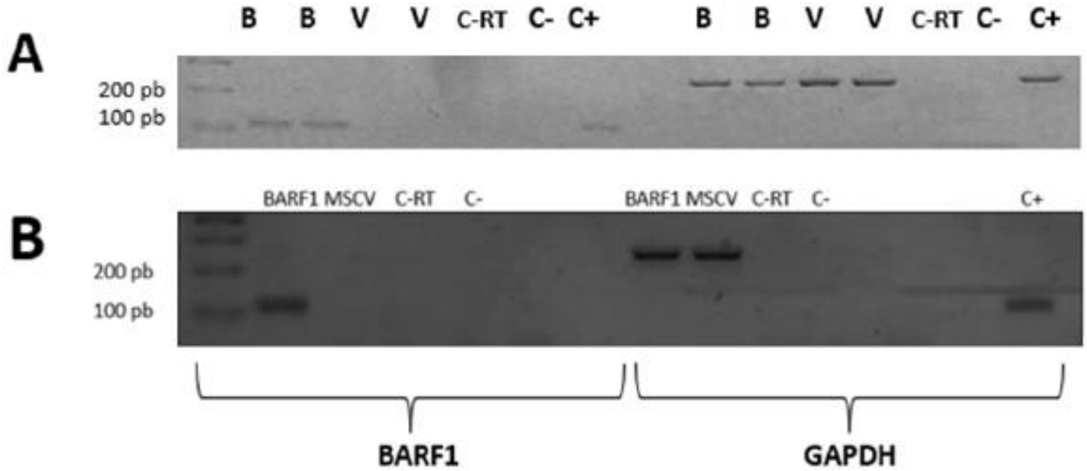

Supplementary figure 3. 2% agarose gel electrophoresis for BARF1 or GAPDH transcript detection by RT-PCR. RNA was isolated from A549 cells (A) and BEAS-2B (B) transfected with MSCVBARF1 construct or an empty vector. (A), B: MSCVBARF1; V: empty vector; C-RT: reverse-transcriptase negative control: C-: Negative control; C+: Positive control (Raji cells). (B), BARF1: MSCVBARF1; MSCV: Empty vector; C-RT: Reverse-transcriptase negative control; C-: Negative control; C+: Positive cont
